# New insights into the influence of pre-culture on robust solvent production of *C. acetobutylicum*

**DOI:** 10.1007/s00253-023-12981-8

**Published:** 2024-01-17

**Authors:** Katharina Oehlenschläger, Marianne Volkmar, Judith Stiefelmaier, Alexander Langsdorf, Dirk Holtmann, Nils Tippkötter, Roland Ulber

**Affiliations:** 1 Institute of Bioprocess Engineering, University of Kaiserslautern-Landau, Gottlieb-Daimler-Straße 49, 67663 Kaiserslautern, Germany; 2 Institute of Bioprocess Engineering and Pharmaceutical Technology, University of Applied Sciences Mittelhessen, Wiesenstraße 14, 35390 Giessen, Germany; 3 Institute of Process Engineering in Life Sciences, Karlsruhe Institute of Technology, Kaiserstraße 12, 76131 Karlsruhe, Germany; 4 Institute of Bioprocess Engineering and Downstream Processing, University of Applied Sciences Aachen, Heinrich-Mußmann-Straße 1, 52428 Jülich, Germany

**Keywords:** Butanol, ABE, *C. acetobutylicum*, Acid crash, Metabolic shift, Pre-culture

## Abstract

**Abstract:**

Clostridia are known for their solvent production, especially the production of butanol. Concerning the projected depletion of fossil fuels, this is of great interest. The cultivation of clostridia is known to be challenging, and it is difficult to achieve reproducible results and robust processes. However, existing publications usually concentrate on the cultivation conditions of the main culture. In this paper, the influence of cryo-conservation and pre-culture on growth and solvent production in the resulting main cultivation are examined. A protocol was developed that leads to reproducible cultivations of *Clostridium acetobutylicum*. Detailed investigation of the cell conservation in cryo-cultures ensured reliable cell growth in the pre-culture. Moreover, a reason for the acid crash in the main culture was found, based on the cultivation conditions of the pre-culture. The critical parameter to avoid the acid crash and accomplish the shift to the solventogenesis of clostridia is the metabolic phase in which the cells of the pre-culture were at the time of inoculation of the main culture; this depends on the cultivation time of the pre-culture. Using cells from the exponential growth phase to inoculate the main culture leads to an acid crash. To achieve the solventogenic phase with butanol production, the inoculum should consist of older cells which are in the stationary growth phase. Considering these parameters, which affect the entire cultivation process, reproducible results and reliable solvent production are ensured.

**Key points:**

• *Both cryo- and pre-culture strongly impact the cultivation of C. acetobutylicum*

• *Cultivation conditions of the pre-culture are a reason for the acid crash*

• *Inoculum from cells in stationary growth phase ensures shift to solventogenesis*

**Graphical Abstract:**

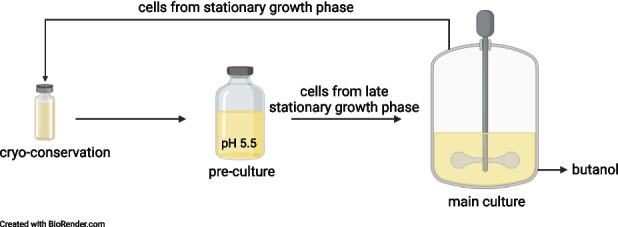

## Introduction

Clostridial fermentation was an extensively used method in the twentieth century to produce acetone, ethanol, and butanol (ABE), but it was successively replaced by a growing petrochemical industry (Jones 2001). However, the projected depletion of fossil resources currently leads to rising oil prices. This results in a newly increasing interest in biological production processes, if possible, based on renewable resources. Butanol, the main product of most solventogenic clostridial fermentations, is an important platform chemical. It is used as a solvent in lacquer or for the formation of more valuable butyl-esters (Mascal 2012). Moreover, the component butanol is viewed as a promising alternative to ethanol as a biofuel because it has very similar physical properties as gasoline and offers a high heating value and energy content (Ibrahim et al. 2017). Therefore, the application of butanol as fuel or fuel additive is feasible in unmodified engines. Also, the ABE mixture was investigated concerning its combustion properties and has the potential for application as a biofuel (Lee et al. 2016).

Saccharolytic clostridia are capable of using a wide variety of carbon sources as substrate, such as cellulose, hemicellulose, and starch after hydrolysis into monomers. This is of special interest as they can use various lignocellulosic biomasses as feedstock (Riaz et al. 2022). Therefore, solvent production with clostridia does not cause the typical food or feed dilemma of first-generation biofuels that use agricultural products.

The most commonly used organism for ABE fermentation is *Clostridium acetobutylicum*. It is a strictly anaerobic, gram-positive bacterium that can be found ubiquitously, but mostly in sediments. As all solventogenic clostridia, *C. acetobutylicum* displays a two-phased metabolism (Monot et al. 1982) and produces the solvents acetone, butanol, and ethanol typically in the molar ratio of 3:6:1 (Jones and Woods 1986). At the first stage of the metabolism, organic acids, mainly butyric acid, are produced. After this so-called acidogenesis, the secreted acids are partly reassimilated and, together with saccharides, used for the formation of solvents in solventogenesis. The particular reason for the onset of the solventogenic phase is not fully resolved yet, but several parameters are connected to the phase shift. These include the pH, the acid concentration, the glucose concentration, and the concentration of nutrients such as nitrogen and phosphate (Kumar et al. 2013). If the cultivation does not enter the solventogenic phase, the produced acids accumulate and lead to a so-called acid crash. This phenomenon is characterised by a drop in the pH and metabolic stagnancy (Maddox et al. 2000). This process is not fully understood yet and is one reason why the cultivation of clostridia is often challenging.

The course of a cultivation depends on various factors such as the available nutrients as well as process parameters like temperature, pH, and agitation. The pre-culture generally also has crucial effects on the growth and productivity of the subsequent main culture and the robustness of the entire process (Becker et al. 2023). Shida et al. examined the influence of the size and growth phase of *Achromobacter delmarvae* and *Escherichia coli* inoculums on the lag phase of the respective main cultures and reported a shorter lag phase for bacteria from the exponential phase (Shida et al. 1977). The adaptation of *E. coli* to a new culture medium during the pre-culture was analysed by Kim et al. (2021). They showed an increased growth rate after sequential transfers to the new medium and proved the presence of genetically adapted cells in the pre-culture. Sandoval et al. investigated the influence of the metabolic phase of the inoculum for *Clostridium beijerinckii* and reported higher growth rates when cells from the exponential growth phase were used as inoculum (Sandoval-Espinola et al. 2015). Moreover, Gutierrez et al. found a connection between the cell motility of the inoculum and the solvent production of *C. acetobutylicum* and suggested using cells with high motility as inoculum (Gutierrez and Maddox 1986).

Despite the crucial effects of the pre-culture on the main culture, its impact is not much investigated yet. This especially applies to the impact of the pre-culture on the product formation in the main culture. Therefore, in this work, the pre-culture has been studied concerning the inoculum, the optimal pH conditions, and the fermentation duration. For all parameters concerning the pre-culture the impact on the main culture was studied. As a result, connections were found between the metabolic phase of the pre-culture at the time of inoculation and the occurrence of the acid crash in the subsequent main culture. By avoiding the acid crash and enabling the shift to the solventogenesis, solvent production in the main culture can be ensured. In this work, a cultivation process of *C.* *acetobutylicum* is presented. The method ensures reliable cell growth and an efficient and robust butanol production.

## Materials and methods

### Microorganism

*Clostridium acetobutylicum* DSM 792 was purchased from DSMZ (Deutsche Stammsammlung für Mikroorganismen und Zellkulturen, German Collection of Microorganisms and Cell Cultures GmbH, Braunschweig, Germany). Cells of *C.* *acetobutylicum* were cryo-conserved in a 40% glycerol solution at – 80 °C under anaerobic conditions.

### Cultivation conditions

*C. acetobutylicum* was cultivated at 37 °C and 50 rpm in serum flasks with a fermentation volume of 150 mL. Anaerobic conditions were ensured by sparging the medium for 40 min with nitrogen. Cryo-cultures consisted of 4 mL cultivation broth mixed with 4 mL of 80% glycerol as a cryo-protective agent. Pre-cultures were inoculated with 1 vol.% of cryo-culture and cultivated in a modified PY + X medium derived from the PY + X medium of DSMZ. Modified PY + X medium contained per liter 5 g tryptone, 10 g yeast extract, 5 g peptone from meat, 5 g glucose, and 40 mL salt solution. Salt solution consisted of 0.25 g CaCl_2_, 0.5 g MgSO_4_ × 7 H_2_O, 1 g KH_2_PO_4_, 1 g K_2_HPO_4_, 10 g NaHCO_3_, and 2 g NaCl per liter. Main cultures were inoculated with 10 vol.% of pre-culture and cultivated in MP2opt medium from Engel et al. MP2opt medium contained per liter 2.2 g ammonium acetate, 0.5 g KH_2_PO_4_, 0.5 g K_2_HPO_4_, 0.2 g MgSO_4_ × 7 H_2_O, 0.015 g Fe(II)SO_4_ × 7 H_2_0, 0.01 g MnSO_4_ × 7 H_2_O, 1 mL of vitamin solution, and 45 g glucose (Engel et al. 2018). Vitamin solution was prepared with 2 mg p-aminobenzoic acid, 2 mg thiamine-HCl, and 0.01 mg D-biotin per liter. The pH of the MP2opt medium was adjusted to 6.8 before inoculation. One-milliliter samples were taken periodically to measure the optical density (OD), pH, and substrate and product concentration. After determining the first two parameters, the sample was centrifuged (centrifuge 5418, rotor FA-45-18-11, Eppendorf SE, Hamburg, Germany) for 2 min at 16,873·g and the supernatant sterile filtered using polyamid filters with a pore size of 0.20 µm (Chromafil Xtra PA20/13, Macherey Nagel, Düren, Germany). Samples were stored at − 20 °C until further analysis.

### Analytical methods

The substrate and product concentration in the supernatant of cultivation samples was analysed using an HPLC system consisting of a Merck Hitachi L-6200 pump (Merck KGaA, Darmstadt, Germany), a Midas cool autosampler (Spark Holland B.V. Emmen, The Netherlands), a Jetstream II plus column thermostat (Duratec, Hockenheim, Germany), an Aminex HPX-87H-column (300 mm × 7.8 mm, Bio-Rad Laboratories GmbH, Feldkirchen, Germany), and a RI detector (PN3140, Postnova, Landsberg am Lech, Germany). The column temperature was 80 °C and the mobile phase consisted of 2.5 mM H_2_SO_4_ at a flow rate of 0.6 mL·min^−1^. Measured values of the glucose concentration were normed to the weighed in concentration. Cell density was measured by the OD_600_ with the photometer Lambda Bio + (Perkin Elmer, Rodgau, Germany). The pH was measured with the pH-meter 221 from Hanna Instruments (Vöhringen, Germany).

## Results

To enable a reliable cultivation of *C.* *acetobutylicum* with a high butanol production, several factors were examined. First, the influence of the cryo-culture was analysed to establish an optimised procedure. This then served as the basis for analysing the influence of the initial pH and the metabolic phase of the pre-culture on the main culture.

### Optimal preparation of cryo-cultures

**Fig. 1:**
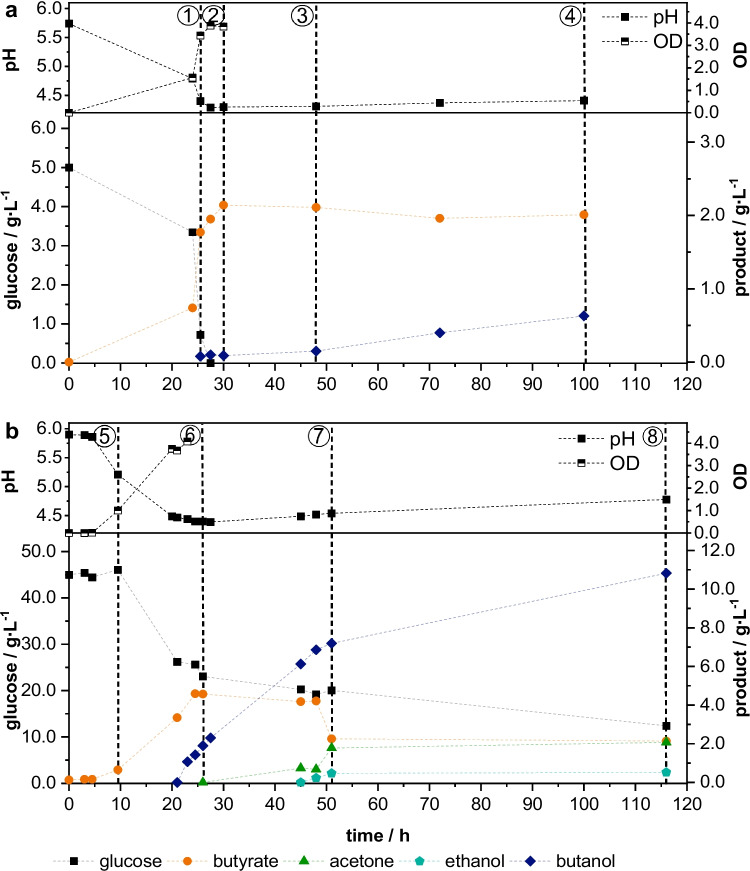
Cultivation of *C.* *acetobutylicum* for the production of cryo-cultures. Dashed lines indicate the time of cell harvest for cryo-conservation, the numbers correspond to the entries in Table 1. **a** Cultivation in PY + X medium, 37 °C, 50 rpm, *V* = 100 mL, inoculum size: 1 vol.%; **b** cultivation in MP2opt medium, 37 °C, 50 rpm, *V* = 150 mL, inoculum size: 10 vol.%; standardized glucose concentration, *n* = 1

Culture degeneration is a known problem of clostridial fermentations, where the strain loses its ability to produce solvents. This is usually the result of subculturing, repeated batch, or continuous fermentation of clostridia that lead to a genetic change (Maddox et al. 2000). A suitable method for cell preservation is therefore of great importance. A common procedure to conserve cell cultures is the preparation of cryo-cultures, where cells are mixed with glycerol and frozen at − 20 to − 80 °C (Julca et al. 2012). Usually, conserving actively growing cells from the exponential growth phase results in fast-growing pre-cultures (Shida et al. 1977). To examine the optimal time for cell extraction, four different points in cultivation time were examined concerning the solvent production in the subsequent cultivation of the conserved cells. Figure 1 shows the cultivation of *C.* *acetobutylicum* in the complex medium PY + X (a) with an initial glucose concentration of 5 g∙L^−1^, which is recommended by DSMZ for the cultivation of *C.* *acetobutylicum*, and in the defined medium MP2opt (b) with initially 45 g∙L^−1^ of glucose, which was optimised for the cultivation of this strain (Engel et al. 2018). Cells were extracted after 25, 30, 48, and 100 h of cultivation in PY + X medium and after 9.5, 26, 51, and 116 h of cultivation in MP2opt medium and used for cryo-conservation. The slight variations in the times of sampling result from the different behaviours of the cells in the different media. This is explained in more detail below. The numbered dashed lines in Fig. 1 indicate the extraction of cells. The hitherto irregular growth behaviour of clostridia prevents the use of cultivation times for the differentiation of the growth phases. Also, the utilisation of the OD is not optimal to detect the shift to the stationary growth phase as cells tend to agglomerate in the synthetic medium, which hampers the OD measurement (Engel et al. 2018). However, the different phases of the clostridial metabolism can be distinguished based on glucose consumption and product formation, which can be monitored based on pH changes (Fig. 1). The exponential growth phase corresponds to the rapid uptake of glucose and the production of acids, which leads to a drop in the pH. In the stationary phase, glucose consumption is limited. In cultivations in which the metabolic shift to solventogenesis takes place, the conversion of butyric acid to ABE products also occurs mainly during the stationary phase. The uptake of acids and their transformation into solvents causes an increase of the pH. The cells were extracted at the end of the exponential growth phase ①, at the shift from exponential to stationary growth phase ②, and at the early ③ and late ④ stationary phases when grown in a complex medium containing 5 g∙L^−1^ glucose (Fig. 1a), and at the start of cell growth ⑤, at the shift of exponential to stationary phase ⑥, and at early ⑦ and late ⑧ stationary phases when grown in defined medium containing 45 g∙L^−1^ glucose (Fig. 1b). Due to the different media compositions, the time to reach the mentioned metabolic phases differs in the two cultivations, which is why the cells from the MP2opt medium were extracted earlier than the ones grown in PY + X medium.

The maximum OD measured in PY + X medium was 3.85. With higher glucose concentration in the MP2opt medium, a maximum OD of 4.1 was measured. Due to the higher amount of available carbohydrates, the cultivation in defined MP2opt medium also results in higher butanol and total solvent concentrations compared to complex PY + X medium. Al-Shorgani et al. established a direct correlation between glucose and ABE concentration up to an initial glucose concentration of 50 g∙L^−1^ (Al-Shorgani et al. 2016). The extracted cells from these cultivations were firstly cryo-conserved for at least 24 h and subsequently used for the inoculation of a pre-culture, with which a cultivation in MP2opt medium was inoculated. This medium is chosen due to its higher glucose content than the PY + X medium. Also, it is an already established medium for main cultivations. Cultivation conditions were the same for all cryo-conservated cells. A pre-culture was inoculated with 1 vol.% of cryo-culture and incubated for 48 h. With these pre-cultures, a main culture was inoculated with 10 vol.%.

Table 1 shows the maximum butanol and total ABE concentration obtained from the subsequent main cultivation in MP2opt medium depending on the time of extraction of the conserved cells. Initial ODs of the main cultures were in a range from 0.32 ± 0.01 which excludes the influence of different cell densities on productivity.

**Table 1:** Effect of cultivation medium and metabolic phase of cells used for cryo-conservation on solvent production in the subsequent fermentation of *C.* *acetobutylicum* cultivated in Mp2opt medium. The course of the fermentation of the cells used for cryo-conservation as well as the sampling time is indicated in Fig. 1, cultivation of pre-culture in PY + X medium: 37 °C, 50 rpm, *V* = 100 mL, inoculum size: 1 vol.%, initial pH = 5.5, fermentation time = 48 h, cultivation of main culture in MP2opt medium: 37 °C, 50 rpm, *V* = 150 mL, inoculum size: 10 vol.%, *n* = 2

Cultivation medium of cells for cryo-conservation	Metabolic phase at time of harvest	Max. butanol in resulting cultivation/g∙L^−1^	Max. ABE in resulting cultivation/g∙L^−1^
PY + X medium	① Late exponential phase	8.15 ± 0.55	11.42 ± 0.60
	② Shift to stationary phase	6.53 ± 2.73	9.22 ± 2.92
	③ Early stationary phase	9.18 ± 0.38	13.16 ± 0.39
	④ Late stationary phase	9.45 ± 0.27	13.6 ± 0.28
MP2opt medium	⑤ Start of cell growth	No cell growth	No cell growth
	⑥ Shift to stationary phase	10.31 ± 0.37	14.65 ± 0.41
	⑦ Early stationary phase	9.84 ± 0.39	14.11 ± 0.51
	⑧ Late stationary phase	11.22 ± 0.30	16.08 ± 0.46

The longer the cells were cultivated before cryo-preservation, the higher the butanol and solvent concentration of the subsequent cultivations. Working with cryo-conservated cells from PY + X medium, cultivations based on cells from the late exponential phase resulted in a maximal butanol concentration of 8.15 g∙L^−1^ and a maximum total solvent concentration of 11.42 g∙L^−1^ in the main culture. These concentrations increased by 16% and 19% to 9.45 and 13.6 g∙L^−1^, respectively, when the cryo-conserved cells originated from the late stationary phase. Cells conserved from MP2opt medium display the same tendency. Here, the butanol and total solvent concentrations increased by 9% and 10%, respectively, and reached 11.22 and 16.08 g∙L^−1^ in the subsequent main culture.

### Influence of the initial pH of the pre-culture

**Fig. 2:**
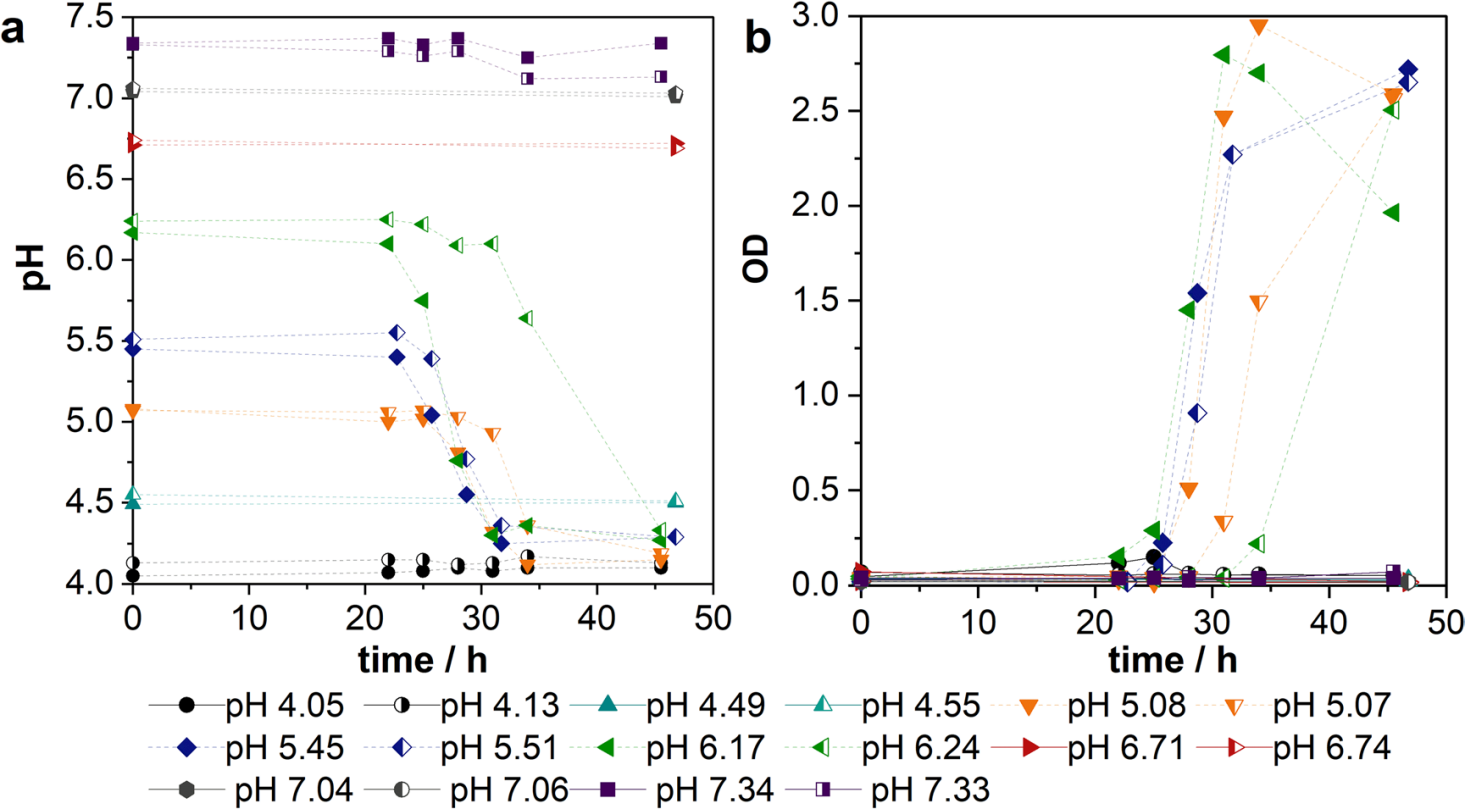
Course of **a** pH and **b** OD in pre-cultures of *C.* *acetobutylicum* cultivated with different initial pH; PY + X medium, 50 rpm, *V* = 100 mL, inoculum size: 1 vol.%, *n* = 1

Based on the preparation of cryo-cultures, which was described previously, the growth of these cells in pre-culture must be ensured. To examine the optimal pH to grow cryo-conservated cells of *C.* *acetobutylicum* in the pre-culture, different initial pH values were adjusted. Figure 2 shows the resulting course of pH and OD in pre-cultures.

For pH values of 6.8 and higher as well as 4.5 and lower, no change in the pH could be observed during a cultivation time of 45 h. This indicates that no acids were produced and therefore no cell growth took place in these cultivations. This is confirmed by the course of the OD (Fig. 2b), where no change can be detected over 48 h. However, for experiments with an initial pH value between 5.0 and 6.2, the pH drops after around 25 h, which indicates the production of acids. In all the growing pre-cultures butyrate was produced in a concentration of 1.91 ± 0.10 g∙L^−1^. Acetate and lactate were produced in lower concentrations of 1.09 ± 0.05 and 0.98 ± 0.18 g∙L^−1^, respectively. The acid production is linked to the exponential growth phase of clostridia, which can also be seen by the increasing OD. The drop of the OD after the exponential growth phase, which can be seen for the pre-cultures with initial pH of 5.08 and 6.17, is a result of the agglomeration of cells, which was observed visually by inspecting the serum flasks.

Above all, the solvent production in the resulting main cultures is of interest. For this reason, all growing pre-cultures were used to inoculate a main culture after a cultivation time of 48 h. Regarding all main cultivations, a good solvent production could be observed: on average a final butanol concentration of 11.46 ± 1.01 g∙L^−1^ was measured. Moreover, a concentration of combined ABE products of 16.27 ± 1.56 g∙L^−1^ was detected.

### Influence of the metabolic phase of the inoculum on product formation

**Fig. 3:**
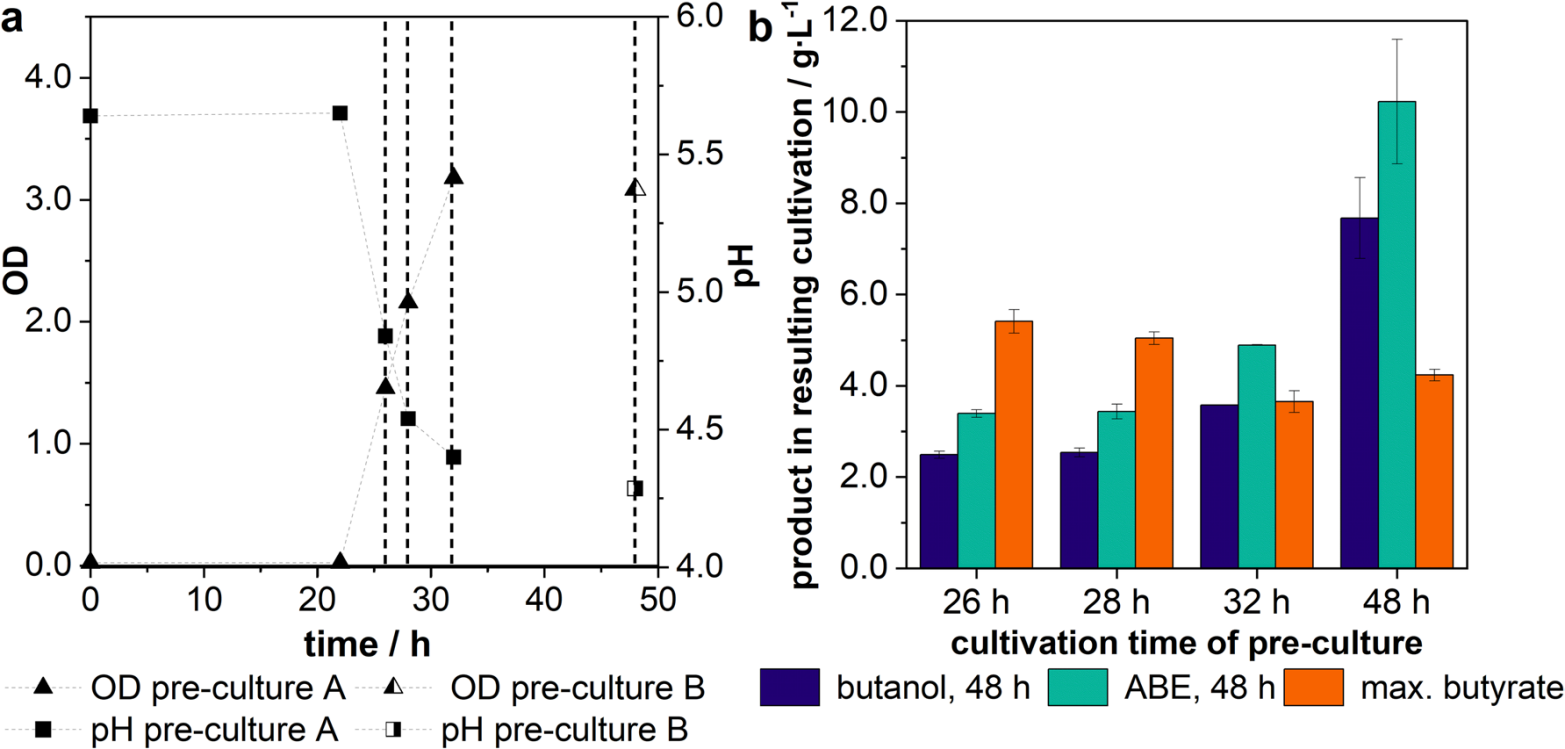
Effect of the cultivation time of the pre-culture on the resulting production in the main culture of *C.* *acetobutylicum* after 48 h of cultivation; **a** OD and pH of pre-cultures used for inoculation, dashed lines indicate extraction time of inoculum; **b** butanol, total ABE, and max. butyrate concentration in main cultures after 48 h inoculated with pre-cultures after different cultivation times; pre-culture: PY + X medium, 50 rpm, *V* = 100 mL, inoculum size: 1 vol.%, pH_0_ = 5.5; main culture: MP2opt medium, 50 rpm, *V* = 150 mL, inoculum size: 10 vol.%, *n* = 2

The growth phase of the pre-culture can influence growth and productivity in the resulting main cultivation. This correlation was investigated based on cultivations that were inoculated with pre-cultures of different cultivation times. The same pre-culture (Fig. 3a, pre-culture A) was used after different cultivation times of 26, 28, and 32 h to inoculate a main culture. As the volume of the pre-culture was limited, an additional pre-culture (Fig. 3a, pre-culture B) with comparable growth behaviour was used as inoculum after a cultivation time of 48 h. A cultivation time of pre-culture of 26 h and 28 h represents cells that were in the exponential growth phase, which was attended by acid production and can be detected by the pH drop. ODs of the pre-culture at the times of 26 h and 28 h were 1.46 and 2.16. At a cultivation time of 32 h, the shift to the stationary growth phase occurred, which was marked by the decline of acid production and the flattening of the pH decrease until it became constant. An OD of 3.18 was reached after a cultivation time of 32 h. At a cultivation time of 48 h acid production and cell growth fully stopped and an OD of 3.08 was measured, which indicated the stationary growth phase. By this approach, cells from the exponential growth phase, the shift to stationary growth phase, and the stationary growth phase were used as inoculum for a main culture. To evaluate the productivity of the resulting main cultivations, the solvent concentration was determined (Fig. 3b). In some cultivations, final solvent concentrations of up to 17.78 g∙L^−1^ were measured, but only after a cultivation time of almost 10 days. This results from a commonly known phenomenon of clostridial fermentations, the so-called “recommencement”, where the acid crash occurs but the acids are degraded slowly and the shift to solventogenesis is possible after an exceptionally long fermentation time (Li et al. 2020; Maddox et al. 2000). This is why the time frame of 48 h was selected here for comparison, to focus on cultivations with high productivity.

A trend to higher solvent production with higher cultivation time of pre-culture is visible. With a cultivation time of the pre-culture of 48 h, the maximal ABE concentration of 10.22 ± 1.36 g∙L^−1^ and a butanol concentration of 7.67 ± 0.88 g∙L^−1^ after 48 h were reached in the main culture. In contrast, in a main culture that was inoculated with a pre-culture of a short cultivation time of 26 h, only 2.48 ± 0.08 g∙L^−1^ of butanol and a total ABE concentration of 3.39 ± 0.08 g∙L^−1^ could be measured. Also, with a cultivation time of 48 h in the pre-culture, a maximal butyrate concentration of 4.23 ± 0.12 g∙L^−1^ was measured in the main culture, which is more than 20% less butyrate compared to a main culture that was inoculated with a pre-culture in the exponential growth phase. A Tukey test was carried out and showed that the resulting butanol concentrations measured from pre-culture cells cultivated for 26, 28, and 32 h significantly differ (*α* < 0.05) to the concentrations measured for pre-culture cells cultivated for 48 h.

**Fig. 4:**
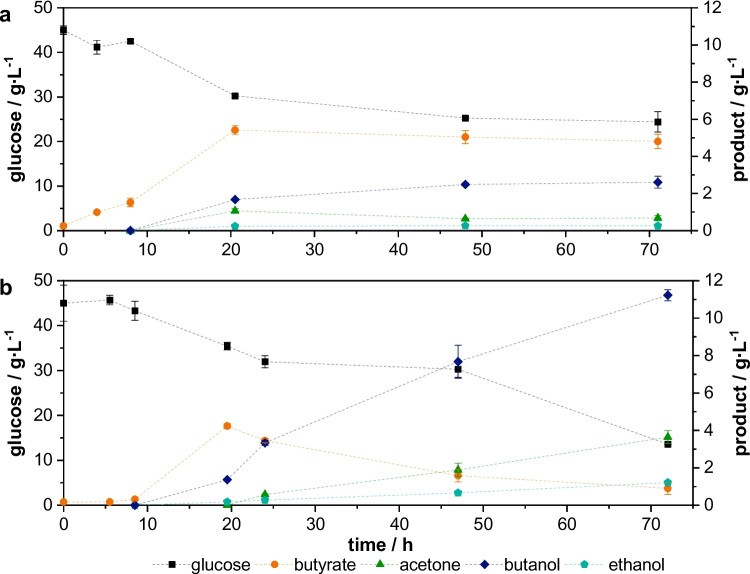
Resulting cultivation of *C.* *acetobutylicum* using pre-cultures with different cultivation times. **a** Cultivation time of pre-culture: 26 h; **b** cultivation time of pre-culture: 48 h; pre-culture: PY + X medium, 50 rpm, *V* = 100 mL, inoculum size: 1 vol.%, pH_0_ = 5,5; main culture: MP2opt medium, 50 rpm, *V* = 150 mL, inoculum size: 10 vol.%; standardized glucose concentration, *n* = 2

For better comparability, the courses of the resulting main cultivation from pre-cultures with short and long cultivation times of 26 and 48 h are exemplarily represented in Fig. 4.

In the resulting cultivation from the pre-culture with a cultivation time of 26 h (Fig. 4a) an “acid crash” was observed, which means that butyrate is produced in high concentrations but no shift to solventogenesis is visible (Maddox et al. 2000). Moreover, the production of butyrate can already be observed after a cultivation time of 4 h, which indicates a very short lag phase. The fast butyrate production leads to a high butyrate concentration of 5.41 ± 0.26 g∙L^−1^ and a low pH of 4.38 ± 0.01. The “acid crash” was also observed in the main culture of the pre-culture with a cultivation time of 28 h. With a longer cultivation time of the pre-culture, the shift to solventogenesis in the main culture occurs (Fig. 4b). In these cultivations, the production of acids starts later at a cultivation time of 9.5 h. Butyrate is produced in lower concentrations and within the solventogenesis, acid is degraded, and the solvent products are built.

## Discussion

With this work, a specific cultivation method for *C.* *acetobutylicum* is examined, which ensures reliable and robust cell growth and the shift to solvent production. Investigations were made regarding the whole cultivation process, including cryo-conservation and pre-cultivation, and the effects of different cultivation conditions on the subsequent main cultivation. The results will be discussed in the following.

The time of cell extraction for cryo-conservation can have an effect on cell growth in the subsequent cultivation (Shida et al. 1977). Besides the growth in pre-cultures, the effect on the solvent production in the main culture is of particular interest, which is why cells of different growth stages were examined concerning the solvent production of resulting main cultures. Here, the cells conserved from a late stationary phase display higher productivity in comparison to cells from earlier cultivation stages. This trend applies for both media tested in this work. The cultivation of cryo-conserved cells from the late stationary phase which were originally grown in MP2opt medium results in a 15% increase in total ABE concentration compared to conserved cells in the same metabolic phase originating from PY + X medium. The statistical significance between the two media was confirmed via a Tukey test (*p* < 0.06). Péter et al. observed higher survival rates of cells from various microorganisms harvested in the stationary phase for cryo-conservation compared to cells from the late exponential phase (Péter and Reichart 2001). For the cultivation of clostridia, there is the additional effect of sporulation, that is initiated by solventogenesis (Ravagnani et al. 2000). The formation of spores due to the solvent production in the defined medium could therefore result in higher stability against the conservation process (Diallo et al. 2021). These findings underline the influence of the metabolic phase in which the cells are conserved for subsequent cultivations and lead to the recommendation to use cells from a late stationary phase grown in MP2opt medium for cryo-preservation.

The pH is a commonly known factor that can influence the growth of bacteria (Stolp et al. 1981). The pH suggested by DSMZ for the cultivation of *C.* *acetobutylicum* in PY + X medium is 6.8–7 (DSMZ). Another commonly used medium for pre-cultures of *C.* *acetobutylicum* is the reinforced clostridial medium, where a pH of 6.6 to 7 is applied (Merck KGaA 2023)*.* Other authors refer to using lower pH for pre-cultivation of different strains of *C.* *acetobutylicum* like 5.8 or 6 (Hartmanis et al. 1986; Li et al. 2011). Based on our investigation, growing pre-cultures could only be observed in a pH range between 5.0 and 6.2. In these cultivations, the OD increased parallel to the decrease of the pH. With a pH of 6.8, which is suggested for clostridia cultivation in PY + X medium by DSMZ, there could not be achieved any cell growth based on cryo-preserved cells in this work. Also important is the effect on solvent production. For all growing pre-cultures, butanol production in a range from 11.46 ± 1.01 g∙L^−1^ was observed, which is near the maximal possible butanol concentration of 14 g∙L^−1^ (Cho et al. 2011). Hence, this work could narrow the initial pH recommended for clostridia down to a range between 5 and 6.2 to achieve reliable growth in the pre-culture. For a pH of 5.5, the fermentation course of independent cultivations was more consistent than for fermentations with a pH closer to the threshold where growth is still possible. This is why a pH of 5.5 is suggested for further cultivations.

The great influence of the metabolic phase of the pre-culture on the solvent production in the main culture was also shown in this work. The butanol and ABE concentration in the main culture after 48 h could be increased by nearly 70% by using a pre-culture of the stationary growth phase instead of a pre-culture of the exponential growth phase. Regarding the increase in cell number with the cultivation time of the pre-culture, it might be reasonably expected that the higher solvent production could be related to a higher cell number. But the fact that there was no increase in cell number after 32 h contradicts this assumption. As the main culture is inoculated using 10 vol.% of pre-culture, not only cells but also metabolites are transferred to the main culture. However, the maximum total acid concentration in the pre-culture is below 5 g∙L^−1^, which leads to a negligible acid concentration in the main culture after the dilution through the inoculation. Also, there is a natural correlation between the composition of the inoculum and the metabolic phase of the pre-culture, which emphasizes the influence of the latter. Furthermore, with an increasing cultivation time of pre-culture, a decrease in butyrate production in the resulting main culture goes along. Hence, the metabolic phase the cells are in during inoculation has a great effect on acid and solvent production in the main culture. This influence is clearly shown in Fig. 4. Cultures inoculated with cells from exponential growth phase result in a short lag phase and rapid acid production, which leads to a low pH. The resulting high concentration of undissociated acids has an inhibitory effect on the metabolic activity (Cho et al. 2011; Gottwald and Gottschalk 1985; Maddox et al. 2000). Sandoval-Espinola et al. investigated the impact of the harvest time of cells of *Clostridium beijerinckii* on the growth of the cells in the resulting culture (Sandoval-Espinola et al. 2015). Harvested cells from the exponential and late exponential growth phases resulted in high maximum specific growth rates (Sandoval-Espinola et al. 2015). This also correlates with the results in this work, as high growth rates are related to high acid production. The rapid acid production then leads to the “acid crash”. Sandoval-Espinola et al. also found that the harvest of cells in the stationary growth phase results in lower specific maximum growth rates in the following culture (Sandoval-Espinola et al. 2015). With this investigation, a longer lag phase and a lower acid production for cultures that were inoculated with cells from stationary growth phase was observed. The lower acid production prevents the occurrence of an acid crash and therefore enables the shift to the solventogenesis. For these cultures, a good solvent production was observed. This enables a reliable solvent production with clostridia even without pH regulation, which is the common method to avoid acid crashes (Capilla et al. 2021).

Based on these findings, the recommended cultivation method for *C.* *acetobutylicum* is described in the following passage: Cryo-cultures should be prepared from cells that were cultivated in MP2opt medium to reach a late stationary phase. The shift to solventogenesis should be ensured because cells from the stationary growth phase display an increased resistance when cryo-conservated, which could be caused by the sporulation. Cryo-cultures were stored in a 40% glycerol solution at − 80 °C. Pre-cultures were then inoculated with 1 vol.% of said cryo-culture. The initial pH range of the pre-culture could be narrowed down to a value between 5.0 and 6.2 and the culture was incubated for approximately 48 h to make sure the stationary growth phase was achieved. This can be detected by following the pH. Main cultures were then inoculated with 10 vol.% of pre-culture. Following this protocol ensured not only a stable growth of the clostridia culture but also reliable solvent production even without external pH regulation. This underlines the importance of the pre-culture for cultivation processes.

## Data Availability

All data supporting the findings of this study are available in the article, supporting information, or upon request from the corresponding author.
